# Progesterone levels on the human chorionic gonadotropin trigger day affect the pregnancy rates for embryos transferred at different stages of development in both general and selected IVF/ICSI populations

**DOI:** 10.1186/s12884-021-03832-3

**Published:** 2021-05-06

**Authors:** P. Merviel, S. Bouée, A. S. Jacamon, J. J. Chabaud, M. T. Le Martelot, S. Roche, C. Rince, H. Drapier, A. Perrin, D. Beauvillard

**Affiliations:** Department of Gynecology, Obstetrics and Reproductive Medicine, Brest University Hospital, 2 avenue Foch, F-29200 Brest, France

**Keywords:** IVF/ICSI, Pregnancy, Progesterone, Blastocyst, Cleaved embryo

## Abstract

**Background:**

Two meta-analyses have shown that pregnancy and birth rates are significantly higher after blastocyst transfer than after cleaved embryo transfer. Other studies have revealed that a serum progesterone level > 1.5 ng/ml on the trigger day is responsible for premature luteinization and is associated with a low pregnancy rate. The objectives of this retrospective study were to determine whether blastocyst transfer gave higher pregnancy rates than cleaved embryo transfer at day 3 in both the general and selected IVF/ICSI populations, and whether the serum progesterone level influenced the pregnancy rate.

**Method:**

We studied IVF/ICSI cycles with GnRH antagonist - FSH/hMG protocols in a general population (*n* = 1210) and a selected “top cycle” population (*n* = 677), after blastocyst transfer on day 5 or cleaved embryo transfer on day 3. The selected couples had to meet the following criteria: female age < 35, first or second cycle, and one or two embryos transferred. We recorded predictive factors for pregnancy and calculated the progesterone to oocyte index (POI), the progesterone:estradiol ratio (P:E2 ratio), and the progesterone to follicle (> 14 mm) index (PFI).

**Results:**

In the general population, the clinical pregnancy rate was significantly higher after blastocyst transfer (33.3%) than after cleaved embryo transfer (25.3%; *p* <  0.01); the same was true for the birth rate (32.1 and 22.8%, respectively, p <  0.01). The differences between blastocyst and embryo transfer groups were not significant in the selected population (respectively 35.7% vs. 35.8% for the clinical pregnancy rate, and 33.9 and 34.9% for the birth rate). The serum progesterone levels on the eve of the trigger day and on the day itself were significantly lower in the pregnant women (*p* <  0.01). We found a serum progesterone threshold of 0.9 ng/ml, as also reported by other researchers. The POI and the PFI appear to have predictive value for cleaved embryos transfers.

**Conclusions:**

Blastocyst transfers were associated with higher clinical pregnancy and birth rates than cleaved embryo transfers in a general population but not in a selected population. The serum progesterone levels on the eve of the trigger day and on the day itself predicted the likelihood of pregnancy.

## Introduction

One in six couples will consult for infertility, defined as failure to achieve a clinical pregnancy after 12 months or more of regular unprotected sexual intercourse [1]. In this setting, in vitro fertilization/intracytoplasmic sperm injection (IVF/ICSI) is one possible treatment option for achieving a live birth. Even though IVF/ICSI has enabled the birth of over 8 million children since 1978, the pregnancy rates per embryo transfer vary from 10 to 50% [2]. Predictive factors for pregnancy include the woman’s age, the cycle rank, and the number of good-quality embryos [3]. In 2013, we defined a select group of “top” IVF/ICSI cycles, with the following characteristics: the first or second IVF/ICSI cycle, female age under 35, and the transfer of no more than two top embryos [4, 5].

Initially, IVF/ICSI was based on cleaved embryo transfer on day 2 (D2) or day 3 (D3). However, progress in in vitro culture and embryo cryopreservation has made it possible to envisage blastocyst transfer on day (D5) or day 6 (D6). Blastocyst transfer has several advantages: decreased uterine exposure to very high serum estradiol levels [6], better synchronization between embryonic development and endometrial maturation, the selection of embryos that progress to a blastocyst stage, and a decrease in uterine contractions [7]. Several meta-analyses have shown that blastocyst transfer is associated with significantly higher clinical pregnancy and birth rates [8, 9]. In some cases, however, cleaved embryos do not develop into blastocysts in vitro, even though the couple may have achieved embryo implantation previously.

The luteal maturation of the endometrium (luteinization) required for embryo implantation depends on the estrogenic effect and adequate progesterone secretion as soon as the ovulatory LH peak has been triggered [10]. In some women in IVF/ICSI, the serum progesterone level peaks too early; this results in premature luteinization and an implantation window that opens (but also closes) earlier. The administration of a gonadotropin releasing hormone (GnRH) antagonist is intended to limit the risk of premature luteinization in IVF/ICSI cycles. A key question in cases of premature luteinization is whether the embryo’s development stage at the time of transfer influences the pregnancy rate. We therefore assessed GnRH antagonist-gonadotropin IVF/ICSI protocols in a general population and a selected population (“top cycles”). The study’s objectives were to (i) measure the pregnancy rates after the transfer of blastocysts or cleaved embryos in these two populations, and (ii) determine whether the serum progesterone level just before the trigger influences the likelihood of pregnancy.

## Material and methods

All the couples enrolled in the IVF/ICSI programs studied here had undergone an infertility assessment. For the women, this included hormone blood tests on the second or third day of the menstrual cycle (including serum levels of FSH, LH, estradiol - E2, prolactin, and anti-Müllerian hormone - AMH), an ultrasound scan of the pelvis (with determination of the antral follicle count), and hysterosalpingography (followed by coelioscopy and/or hysteroscopy, if the results were abnormal). For the men, the assessment included a semen analysis and a sperm motility test. All the hormone blood tests and sperm examinations were performed in the same laboratory.

The main inclusion criteria were physician-diagnosed infertility, female age 18 to 42, male age 18 to 59, and written consent to infertility treatment. Given that the couples had already consented to exploitation of their personal medical data for research purposes, and in line with the French legislation on analyses of routine medical care, approval by an independent ethics committee was not required. The main exclusion criteria were ovarian insufficiency (antral follicle count < 5, AMH < 0.5 ng/ml, and/or FSH > 15 IU/l), severe abnormality of the uterine cavity (synechiae or diffuse adenomyosis), irreversible azoospermia (according to a testicular biopsy), and globozoospermia. We excluded cycles involving donor gametes or donor embryos.

In a controlled ovarian stimulation protocol with a GnRH antagonist, stimulation was initiated on D2 of the cycle with FSH (Fostimon®, Genevrier, Sophia-Antipolis, France/ Gonal-F®, Merck, Lyon, France/ Puregon®, MSD, Levallois-Perret, France) or hMG (Menopur®, Ferring SAS, St Prex, Switzerland), together with 0.25 mg per day ganirelix (Orgalutran®, MSD, Levallois-Perret, France) when the follicle size exceeded 14 mm or the estradiol (E2) level was over 400 pg/mL. The gonadotropin doses were adjusted after the first ultrasound examination and the E2 and LH assays on gonadotropin D5. When at least three follicles had reached a diameter ≥ 17 mm, a dose of 250 μg of recombinant human chorionic gonadotropin (Ovitrelle®, Merck, Lyon, France) was administered. Oocytes (O) were retrieved 35 h after hCG administration.

Luteal phase supplementation consisted of 400 mg per day of intravaginal micronized progesterone (Utrogestan®; Besins International, Paris, France) from the evening of oocyte retrieval until the β-hCG assay two weeks later. Our center applies a freeze-all strategy if (i) the serum progesterone level on the hCG trigger day exceeds 1.5 ng/ml, (ii) ovarian hyperstimulation (> 20 follicles) is observed on ultrasound or the serum estradiol level exceeds 3500 pg/ml on the hCG trigger day, or (iii) spermatozoids are not available on the day of the oocyte retrieval.

For the ICSI procedure, the corona radiata as removed mechanically under a dissecting microscope, and the cumulus was exposed to 0.5% hyaluronidase (Sigma-Aldrich, St. Louis, MO, USA) for 30 s. The partner’s sperm was analyzed according to the WHO classification [11] and then prepared for ICSI. The oocytes were examined after 18 h of incubation at 37 °C in a humidified atmosphere with 5% CO_2_; the presence of two pronuclei (2PN) was a sign of fertilization. The fertilization rate was defined as the ratio between the number of fertilized (2PN) oocytes and the number of mature (metaphase II) oocytes. The resulting embryos were cultured up until D3 or D5. The cleavage rate corresponds to the ratio between D3 cleaved embryos and mature oocytes. Moreover, we recorded the embryos’ frequency of progression from D3 to D5 (i.e. the blastocyst/embryo ratio). A suitable cleaved embryo on D3 was defined as the presence of 8 to 14 uniform-size blastomeres, a fragmentation rate below 50%, and the absence of multinucleation [12]. Blastocysts were classified according to Gardner’s criteria [13]. Adequate blastocyst quality on D5 was defined as stage B3 to B6, an inner cell mass, and fragmentation types AA, AB, BA, BB and (very occasionally) CA and CB, and the absence of multinucleation. Embryos were transferred alternately on either D3 or D5 because the study’s objective was to evaluate the pregnancy rate under these two circumstances. One or two of the best embryos were transferred in utero using a Frydman catheter (CCD Laboratories, Paris, France), and other good-quality embryos were cryopreserved.

Clinical pregnancy was confirmed by ultrasound and a β-hCG level above 1000 IU/L 6 to 8 weeks after embryo transfer, and the clinical pregnancy rate was calculated relative to the number of cycles with a transfer. An ongoing pregnancy was defined as a pregnancy after more than 12 weeks of amenorrhea (WA). The miscarriage rate was calculated with regard to the number of clinical pregnancies after the first trimester, and a miscarriage was classified as pregnancy loss before 12 WA. A live birth was defined as a healthy child birth later than 22 week of gestation. Each couple assessed in the present study went through a single ICSI cycle.

The study’s primary outcome measure was the clinical pregnancy, ongoing pregnancy and live birth rates for cleaved embryo transfers on D3 vs. blastocyst transfers on D5. Starting from the general population, we selected a subpopulation of “top cycles”, as described previously [4, 5]. In the “top cycle”, the couples were in their first or second IVF/ICSI cycle, the women was under the age of 35, and one or two top embryos were transferred. In each group, we looked for predictors of pregnancy and analyzed the pregnancy rates above and below the progesterone threshold of 0.9 ng/ml defined by Venetis et al. [14]. The idea to study a selected population (“top cycle”) is to check pregnancy rates by embryo stage and the impact of the progesterone levels in a population of good prognosis. In particular, do the young age of women, the notion of a first or second cycle and the good quality of the embryo change the results observed in the general population.

Based on the serum progesterone (P) level on the trigger day, we calculated three ratios already described in the literature, which may explain the impact of the progesterone on the occurrence of pregnancy: P (in ng/ml) divided by the number of oocytes retrieved (O), so called as progesterone to oocyte index (POI) [15], P (in pg/ml) divided by the serum E2 level (in pg/ml) on the trigger day (P:E2 ratio [16];), and P (in nM) divided by the number of follicles > 14 mm (a ratio referred to as the progesterone to follicle index (PFI) in the literature [17]). A progesterone concentration of 1 ng/ml corresponds to 3.18 nM.

### Statistical analysis

Continuous variables were expressed as the mean ± standard deviation (SD) (for normally distributed data) or the median [interquartile range]. Categorical variables were expressed as the frequency (percentage). Differences between groups were probed with Student’s t test or Pearson’s chi-squared test. All analyses were performed using Microsoft® Excel (Microsoft Corporation, Redmond, WA, USA) and the XLSTAT® add-on (Addinsoft, Paris, France). The threshold for statistical significance was set to *p* <  0.05. Given that we extracted data from electronic medical records (Medifirst®, Montigny le Bretonneux, France), none of the participants’ clinical and laboratory data were missing.

## Results

We analyzed 1210 IVF/ICSI cycles (general population) with a GnRH antagonist - FSH/hMG protocol between 2016 and 2018 in our ART center of the Brest University Hospital. These cycles resulted in 1156 oocyte retrievals (95.5% per cycle) and 849 embryo transfers (73.4% per retrieval). The demographic, clinical and laboratory data on the general population are summarized as a function of the presence or absence of a pregnancy and by embryo transfer stage in Table 1. With regard to the demographic variables, the various groups did not differ in term of female age, female and male BMIs, primary infertility, the etiology of the infertility, the AMH level on D3 of the menstrual cycle, and the cycle rank. It is noteworthy that smoking was significantly more frequent among the pregnant women. During ovarian stimulation, the serum progesterone level was significantly higher on the eve of the hCG day and on the day itself among the non-pregnant women. We also noted a difference in the serum LH level as a function of pregnancy, depending on the transfer stage; the LH level was higher among pregnant women than non-pregnant women in the blastocyst transfer group but lower among pregnant women than non-pregnant women in the cleaved embryo transfer group. Among the non-pregnant women, the fertilization rate was significantly lower in the cleaved embryo transfer group (*p* <  0.02). Two hundred and forty-two clinical pregnancies were obtained (28.5% per transfer), including 112 after blastocyst transfers (33.3% per transfer; implantation rate (IR): 27.7%) and 130 after cleaved embryo transfers (25.3% per transfer; IR: 17.2%; *p* <  0.01). We recorded 99 ongoing pregnancies (29.4% per transfer) and 108 live births (32.1% per transfer; 9 of the births (8.2%) corresponded to twin pregnancies) after blastocyst transfer, and 112 ongoing pregnancies (21.8% per transfer; *p* <  0.02) and 117 live births (22.8% per transfer; *p* <  0.01; 5 of the births (3.8%) corresponded to twin pregnancies) after cleaved embryo transfer.

**Table 1:** Demographic, clinical and laboratory data for the general population after blastocyst or cleaved embryo transfer. Analysis between pregnant and not pregnant women

Variables	Blastocyst	Blastocyst	p	Cleaved embryo	Cleaved embryo	p
	Pregnancy	No pregnancy		Pregnancy	No pregnancy	
Female age (y.o)	33.8 ± 6.8	33.4 ± 7.8	NS	33.1 ± 7.4	33.7 ± 9.2	NS
Female BMI (kg/m^2^)	24.8 ± 4.5	25.0 ± 5.0	NS	25.2 ± 4.7	24.9 ± 4.2	NS
Female tobacco %	50.0 ^c^	32.0 ^g^	< 0.001	35.0 ^d^	24.6 ^h^	< 0.05
Male BMI (kg/m^2^)	25.1 ± 3.3	25.2 ± 3.8	NS	25.4 ± 7.3	24.8 ± 8.7	NS
Male tobacco %	51.8	33.0 ^c^	< 0.001	43.8	44.0 ^d^	NS
Primary infertility %	65.0	60.8	NS	59.6	62.5	NS
Indications %						
Tubal disorder	16.2	17.9	NS	15.7	13.9	NS
Endometriosis	6.3	7.7	NS	7.1	6.0	NS
Ovulatory dysfunction	6.8	7.0	NS	3.5	4.6	NS
Low ovarian reserve	5.6	6.0	NS	10.7	10.7	NS
Unexplained	14.5	9.9	NS	11.4	8.9	NS
Male factor	28.7	33.6	NS	35.7	35.0	NS
Mixed	21.8	17.9	NS	15.7	21.0	NS
AMH, ng/ml	3.46 ± 2.41 ^a^	3.05 ± 2.70 ^a^	NS	1.95 ± 0.66 ^b^	2.07 ± 1.10 ^b^	NS
Retrieval rank (m ± SD)	1.05 ± 0.9	1.14 ± 0.9 ^e^	NS	1.17 ± 1.0	1.34 ± 1.3 ^f^	NS
Number of cycles	160	312		192	546	
Total dose of Gnt (IU)	2222 ± 819 ^a^	2281 ± 633 ^a^	NS	2727 ± 664 ^b^	3127 ± 674 ^b^	< 0.001
FSH use, %	57.5	58.3	NS	65.1	63.5	NS
Stimulation duration (d)	10.06 ± 1.60	9.82 ± 1.49 ^c^	NS	10.07 ± 1.79	10.18 ± 1.76 ^d^	NS
Mature follicle hCG day	3.4 ± 2.0	4.1 ± 2.7 ^a^	< 0.01	3.2 ± 1.8	2.5 ± 2.1 ^b^	< 0.001
Estradiol hCG day-2	1161 ± 530	1269 ± 528	< 0.05	1004 ± 335	1093 ± 460	< 0.05
Estradiol hCG day-1	1677 ± 607	1750 ± 723	NS	1472 ± 398	1464 ± 675	NS
Estradiol hCG day	1973 ± 613 ^a^	1997 ± 689 ^a^	NS	1667 ± 531 ^b^	1733 ± 722 ^b^	NS
Progesterone hCG day-2	0.73 ± 0.21	0.75 ± 0.30	NS	0.65 ± 0.17	0.79 ± 0.26	< 0.001
Progesterone hCG day-1	0.78 ± 0.29	0.90 ± 0.29	< 0.001	0.75 ± 0.27	0.84 ± 0.32	< 0.01
Progesterone hCG day	0.84 ± 0.22	0.99 ± 0.36 ^a^	< 0.001	0.83 ± 0.22	0.91 ± 0.31 ^b^	< 0.01
LH hCG day-2	2.17 ± 1.32	1.43 ± 1.23	< 0.001	1.68 ± 1.19	1.77 ± 1.32	NS
LH hCG day-1	1.73 ± 1.19	1.34 ± 1.28	< 0.01	1.37 ± 0.76	1.88 ± 1.65	< 0.001
LH hCG day	1.80 ± 1.47	1.49 ± 1.34 ^a^	< 0.05	1.61 ± 0.77	1.90 ± 1.27 ^b^	< 0.01
Endometrium (mm)	9.51 ± 1.49 ^e^	9.62 ± 1.99	NS	9.98 ± 2.08 ^f^	9.60 ± 1.86	NS
Stimulation cancelled	6 (3.7%)	12 (3.8%)	NS	8 (4.1%)	28 (5.1%)	NS
Number oocyte retrievals	154	300		184	518	
Retrieved oocytes (m ± SD)	9.10 ± 3.73 ^a^	9.64 ± 4.09 ^a^	NS	6.77 ± 2.68 ^b^	6.43 ± 3.03 ^b^	NS
Mature oocytes (m ± SD)	7.54 ± 2.73 ^a^	7.84 ± 3.41 ^a^	NS	5.07 ± 2.31 ^b^	4.71 ± 2.47 ^b^	NS
ICSI, %	57.1 ^a^	57.0 ^a^	NS	64.6 ^b^	69.1 ^b^	NS
2PN (m ± SD)	5.69 ± 2.26 ^a^	5.87 ± 2.77 ^a^	NS	3.56 ± 2.11 ^b^	2.96 ± 1.85 ^b^	< 0.01
Fertilization rate, %	75.5	74.9 ^a^	NS	70.2	62.5 ^b^	< 0.02
Total embryos (m ± SD)	5.75 ± 2.47 ^a^	6.02 ± 2.68 ^a^	NS	3.54 ± 1.86 ^b^	3.08 ± 1.75 ^b^	< 0.02
Cleavage rate, %	77.6	76.4 ^a^	NS	69.8	65.4 ^b^	NS
Blastocyst (m ± SD)	2.71 ± 1.74	2.62 ± 1.77	NS	–	–	
% Blastocyst/embryo	47.2	43.6	NS	–	–	
Culture failure	42 (27.2%)	76 (25.3%)	NS	54 (29.3%)	135 (26.0%)	NS
Number of transfers	112	224		130	383	
Embryos transferred (m ± SD)	1.33 ± 0.94 ^c^	1.30 ± 0.92 ^c^	NS	1.59 ± 0.40 ^d^	1.51 ± 0.89 ^d^	NS

Legends: IU: international unit; Gnt: gonadotropin; endometrium: endometrial thickness

Statistical analysis: The “p” column indicates the difference between pregnant and not pregnant women in cases of blastocyst or cleaved embryo transfer

Difference between blastocyst and cleaved embryo transfers: ^a-b^: *p* <  0.001, ^c-d^: *p* <  0.01, ^e-f^: *p* <  0.02, ^g-h^: *p* <  0.05

**Fig. 1:**
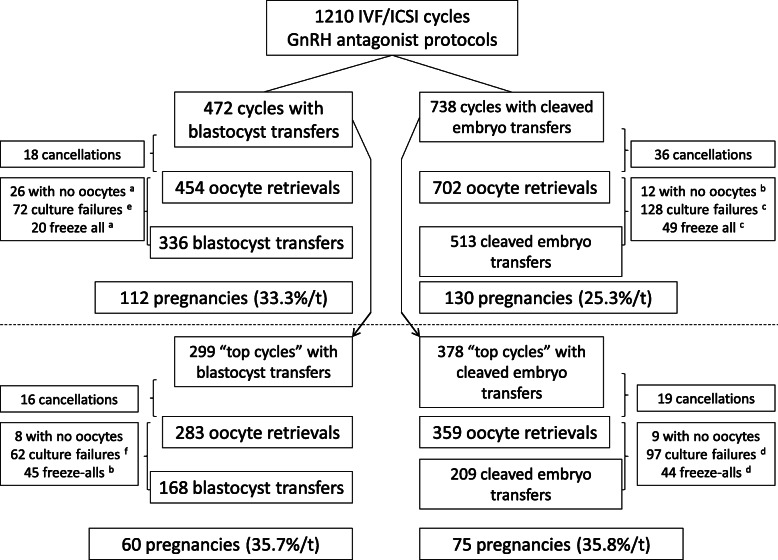
Study flow chart for the general population (above the dotted line) and the selected population (below the dotted line). E: embryo; t: transfer; culture failures: includes fertilization and embryo culture failures, and poor embryo quality. Statistical analysis: Difference between blastocyst and cleaved embryo transfers: ^a-b^: *p* < 0.001; ^c-d^: *p* < 0.01; ^e-f^: *p* < 0.05

Of the 1210 cycles, 677 (55.9%) met the “top cycle” criteria. They resulted in 642 oocyte retrievals (94.8% per cycle) and 377 embryo transfers (58.7% per retrieval). The demographic, clinical and laboratory data in the “top cycle” selected population are summarized as a function of the presence or absence of a pregnancy and by embryo transfer stage in Table 2. With regard to the demographic variables, the groups did not differ significantly other than for the cycle rank (low in the women who became pregnant after blastocyst transfer and high in the women who became pregnant after cleaved embryo transfer). During ovarian stimulation, the serum progesterone level was significantly higher on the eve of the hCG day and on the day itself among the non-pregnant women. We also found a difference in the serum LH level, which was significantly higher among the pregnant women than the non-pregnant women in both the blastocyst transfer and cleaved embryo transfer groups. It is noteworthy that the clinical pregnancy rate in women aged ≥35 and/or with 3 or more cycles was 30.9% after blastocyst transfer (*n* = 168) and 18% after cleaved embryo transfer (*n* = 304) (data not shown). One hundred and thirty-five clinical pregnancies were obtained (35.8% per transfer), including 60 after a blastocyst transfer (35.7% per transfer; IR: 32.4%) and 75 after a cleaved embryo transfer (35.8% per transfer; IR: 30.9%; p = not significant - NS; Fig. 1). We observed 55 ongoing pregnancies (32.7% per transfer) and 57 live births (33.9% per transfer; 1 of the births (1.6%) corresponded to a twin pregnancy) after blastocyst transfer, and 69 ongoing pregnancies (33.0% per transfer; p = NS) and 73 live births (34.9% per transfer; p = NS; 4 of the births (5.3%) corresponded to twin pregnancies) after cleaved embryo transfer.

**Table 2:** Demographic, clinical and laboratory data for the “top cycle” selected population after blastocyst transfers or cleaved embryo transfer. Analysis between pregnant and not pregnant women

Variables	Blastocyst	Blastocyst	p	Cleaved embryo	Cleaved embryo	p
	Pregnancy	No pregnancy		Pregnancy	No pregnancy	
Female age (y.o)	30.7 ± 2.4	31.0 ± 2.8	NS	30.4 ± 2.5	30.3 ± 2.3	NS
Female BMI (kg/m^2^)	25.2 ± 4.5	24.7 ± 2.1	NS	25.7 ± 4.9	24.8 ± 4.1	NS
Female smoking, %	26.7	19.5 ^e^	NS	31.2	29.9 ^f^	NS
Male BMI (kg/m^2^)	25.1 ± 3.3	25.3 ± 4.3	NS	25.3 ± 4.0	25.2 ± 4.2	NS
Male smoking, %	34.2	28.3 ^g^	NS	38.7	38.0 ^h^	NS
Primary infertility, %	40.0 ^c^	43.8 ^a^	NS	61.0 ^d^	66.8 ^b^	NS
Indications %						
Tubal disorder	16.1	15.0	NS	15.2	13.7	NS
Endometriosis	6.6	8.2	NS	7.6	6.8	NS
Ovulatory dysfunction	8.5	11.8	NS	3.8	4.8	NS
Low ovarian reserve	6.6	5.1	NS	9.1	8.5	NS
Unexplained	11.4	10.8	NS	10.6	9.7	NS
Male factor	30.4	30.4	NS	37.4	38.4	NS
Mixed	20.4	18.8	NS	16.0	17.8	NS
AMH ng/ml	3.70 ± 2.19	4.10 ± 3.60	NS	3.41 ± 1.99	3.70 ± 3.39	NS
Retrieval rank (m ± SD)	0.53 ± 0.50 ^g^	0.65 ± 0.47 ^e^	< 0.05	0.67 ± 0.46 ^h^	0.54 ± 0.50 ^f^	< 0.02
Number of cycles	105	194		131	247	
Total dose Gnt (IU)	2125 ± 721	1913 ± 670	< 0.02	2203 ± 853	1965 ± 813	< 0.01
FSH use, %	34.2 ^a^	39.1 ^a^	NS	64.8 ^b^	61.9 ^b^	NS
Stimulation duration (d)	10.0 ± 1.58	10.22 ± 2.96	NS	10.1 ± 1.5	10.1 ± 2.7	NS
Mature follicle hCG day	2.52 ± 2.25	3.11 ± 2.98 ^c^	NS	2.82 ± 2.72	2.22 ± 2.00 ^d^	< 0.05
Estradiol hCG day-2	1155 ± 586	1264 ± 513	NS	1142 ± 584	1235 ± 513	NS
Estradiol hCG day-1	1624 ± 630	1706 ± 593	NS	1601 ± 584	1608 ± 681	NS
Estradiol hCG day	1899 ± 573	2039 ± 628	NS	1832 ± 583	2012 ± 665	< 0.01
Progesterone hCG day-2	0.75 ± 0.21	0.76 ± 0.29	NS	0.76 ± 0.23	0.75 ± 0.28	NS
Progesterone hCG day-1	0.78 ± 0.26	0.89 ± 0.27	< 0.001	0.78 ± 0.28	0.87 ± 0.28	< 0.01
Progesterone hCG day	0.86 ± 0.21	0.99 ± 0.39	< 0.001	0.86 ± 0.21	1.00 ± 0.39	< 0.001
LH hCG day-2	2.73 ± 1.86	1.61 ± 1.39	< 0.001	2.66 ± 1.72	1.55 ± 1.31	< 0.001
LH hCG day-1	1.42 ± 0.81	1.34 ± 1.16	NS	1.51 ± 0.83	1.41 ± 1.27	NS
LH hCG day	2.15 ± 1.30	1.58 ± 1.47	< 0.001	2.25 ± 1.31	1.68 ± 1.43	< 0.001
Endometrium (mm)	9.63 ± 1.47	9.54 ± 1.78	NS	9.66 ± 1.51	9.53 ± 1.75	NS
Stimulation cancelled	5 (4.7%)	11 (5.6%)	NS	6 (4.5%)	13 (5.2%)	NS
Number oocyte retrievals	100	183		125	234	
Retrieved oocytes (m ± SD)	8.73 ± 3.25	9.89 ± 3.74	< 0.01	8.16 ± 3.24	9.26 ± 3.81	< 0.01
Mature oocytes (m ± SD)	7.38 ± 2.64	7.96 ± 3.06	NS	6.85 ± 2.71	7.35 ± 3.19	NS
ICSI, %	34.6 ^a^	32.9 ^a^	NS	57.3 ^b^	53.7 ^b^	NS
2 PN (m ± SD)	5.40 ± 2.40	6.06 ± 2.68 ^c^	< 0.05	4.90 ± 2.44	5.36 ± 2.87 ^d^	NS
Fertilization rate, %	74.3	76.1	NS	72.5	72.9	NS
Total embryos (m ± SD)	5.18 ± 2.35	6.15 ± 2.55 ^c^	< 0.01	4.94 ± 2.45	5.48 ± 2.71 ^d^	NS
Cleavage rate, %	75.4	77.3	NS	73.1	74.5	NS
Blastocyst (m ± SD)	2.93 ± 1.77	3.25 ± 1.91	NS	–	–	
% Blastocyst/embryo	53.5	52.7	NS	–	–	
Culture failure	40 (40.0%)	75 (40.9%)	NS	50 (40.0%)	100 (42.7%)	NS
Number of transfers	60	108		75	134	
Embryo transferred (m ± SD)	1.15 ± 0.34	1.13 ± 0.33	NS	1.17 ± 0.37	1.14 ± 0.35	NS

Legends: IU: international unit; Gnt: gonadotropin; endometrium: endometrial thickness

Statistical analysis: The “p” column indicates the difference between pregnant and not pregnant women in cases of blastocyst or cleaved embryo transfer

Difference between blastocyst and cleaved embryo transfers: ^a-b^: *p* < 0.001, ^c-d^: *p* < 0.01, ^e-f^: *p* < 0.02, ^g-h^: *p* < 0.05

The lower transfer rates per retrieval were due to the significantly higher number of culture failures and freeze-all procedures for the “top cycle” population in both the blastocyst transfer and cleaved embryo transfer groups (Fig. 1). In our study, 2.1% of the cycles had a progesterone level ≥ 1.5 ng/ml on the trigger day (range: 1.5 to 2.9). With regard to the 69 planned cases of “freeze all” in the general population, 13 couples did not have any embryos frozen, and the other 56 had a mean of 2.96 ± 2.03 embryos frozen. In the “top cycle” selected population, 6 couples in each group (i.e. the blastocyst transfer group and the cleaved embryo transfer group) did not have any frozen embryos, and the others obtained 3.43 ± 2.14 (range: 1–10) and 3.44 ± 2.11 (range: 1–10) frozen embryos, respectively. In our ART center, the pregnancy rates after frozen embryo transfer have been above 20% for the last few years, so we expect to obtain good cumulative pregnancy rate (for fresh + frozen transfers). In the general population, the retrieval failure rate (no oocytes) was significantly higher in the blastocyst group transfer group (5.7%) than in the cleaved embryo transfer group (1.7%; *p* <  0.001).

The calculated POI, P:E2 and PFI ratios are given in Table 3. The POI differed significantly according to the type of transfer in the general population, and was significantly lower in the selected population than in the general population. The P:E2 ratio did not differ significantly - probably because it was well below the threshold for premature luteinization. All the PFI values were significantly higher (*p* <  0.001) in the selected population than in the general population, and there was a significant difference (p <  0.001) between pregnant and non-pregnant women in the cleaved embryo transfer group.

**Table 3:** Results for the various index/ratios involving the serum progesterone level on the hCG trigger day. Analysis between pregnant and not pregnant women, blastocyst or cleaved embryo transfers, and general or selected populations

Variables	Blastocyst Pregnancy	Blastocyst No pregnancy	p	Cleaved embryo Pregnancy	Cleaved embryo No pregnancy	p
General population						
cycles: n	160	312		192	546	
POI (ng/ml/oocyte)	0.092 ± 0.058	0.102 ± 0.068	NS	0.122 ± 0.072^c^	0.141 ± 0.082^a^	< 0.01
P:E2 (pg/ml/pg/ml)	0.42 ± 0.35	0.49 ± 0.32	< 0.05	0.49 ± 0.31	0.52 ± 0.32	NS
PFI (nmole/l/follicle)	0.78 ± 0.34^a^	0.76 ± 0.42^a^	NS	0.82 ± 0.38^a^	1.15 ± 0.46^a^	< 0.001
Selected population						
cycles: n	105	194		131	247	
POI (ng/ml/oocyte)	0.098 ± 0.064	0.100 ± 0.084	NS	0.105 ± 0.065^d^	0.107 ± 0.072^b^	NS
P:E2 (pg/ml/pg/ml)	0.45 ± 0.36	0.48 ± 0.32	NS	0.46 ± 0.36	0.49 ± 0.38	NS
PFI (nmole/l/follicle)	1.08 ± 0.29^b^	1.01 ± 0.41^b^	NS	0.96 ± 0.24^b^	1.43 ± 0.62^b^	< 0.001

Legends: *POI* progesterone to oocyte index, *P:E2* progesterone to estradiol ratio, *PFI* progesterone to follicle index, *NS* not significant

Statistical analysis: The “p” column indicates the difference between pregnant and not pregnant women in cases of blastocyst or cleaved embryo transfer

Difference between general and selected population (“top cycles”) for each index or ratio after blastocyst or cleaved embryo transfers: ^a-b^*p* < 0.001, ^c-d^*p* < 0.05

**Fig. 2:**
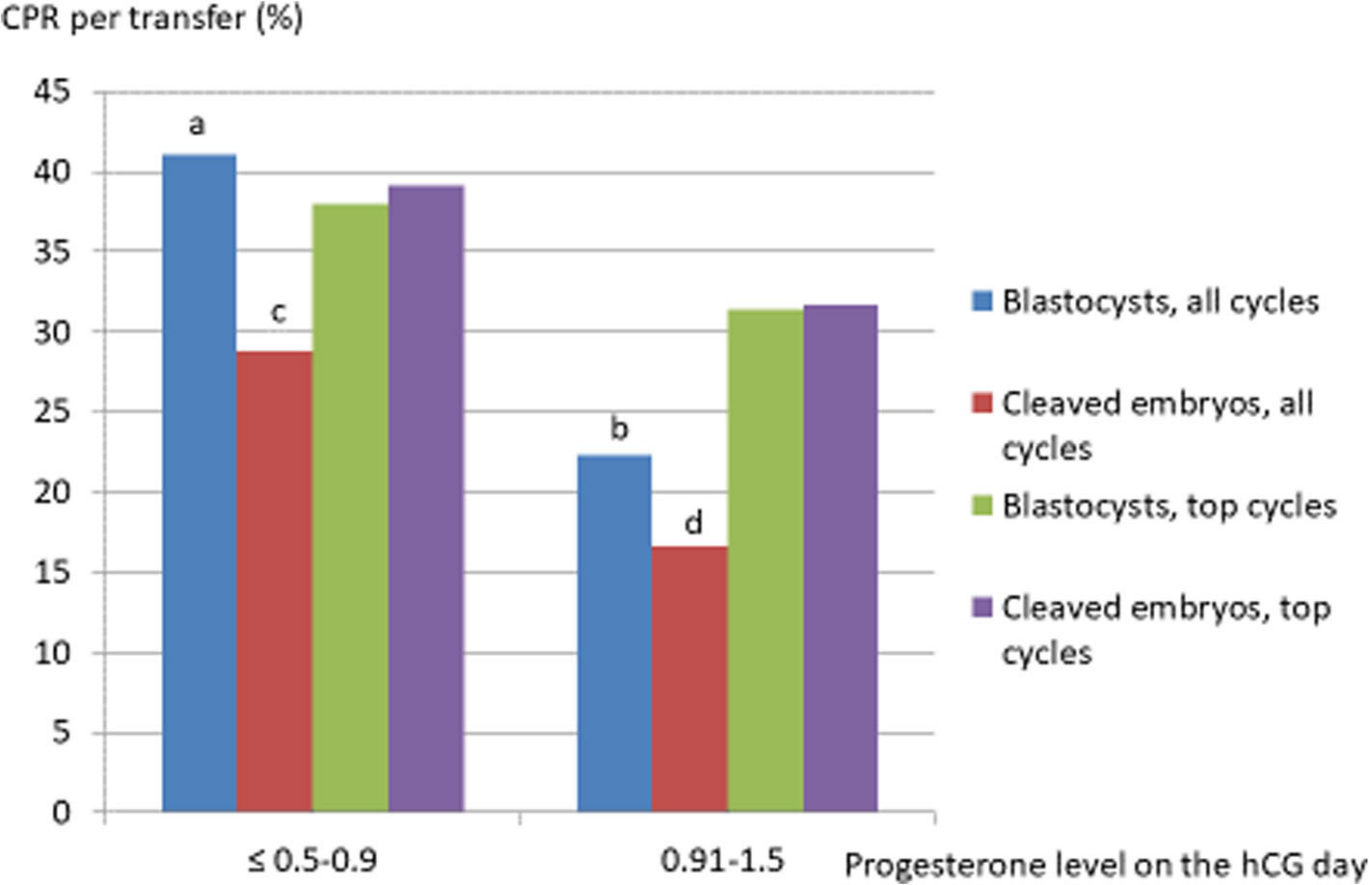
Clinical pregnancy rates above and below the progesterone threshold (≤ 0.9 and > 0.9 ng/ml), after blastocyst or cleaved embryo transfers in general (all cycles) and selected (top cycles) populations. CPR: clinical pregnancy rate. Statistical analysis: ^a-b^: *p* < 0.02; ^c-d^: *p* < 0.05

**Fig. 3:**
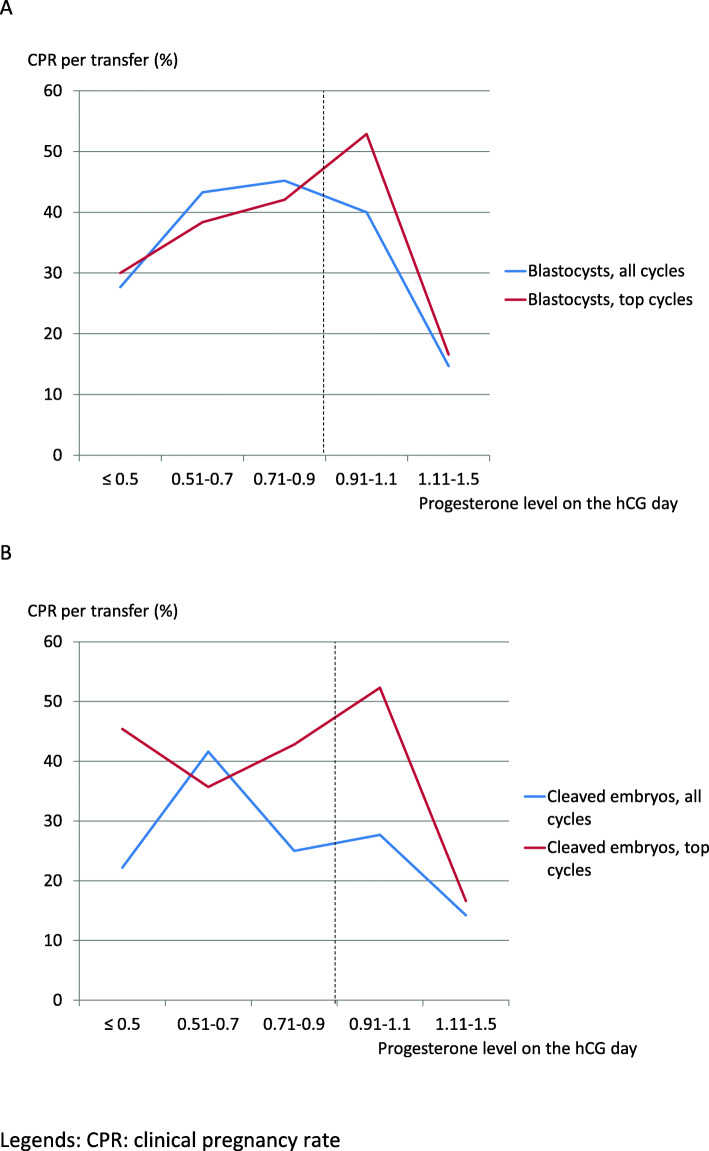
Clinical pregnancy rates as a function of the progesterone range (ng/ml) after blastocyst transfers (**a**) and cleaved embryo transfers (**b**), in general (all cycles) and selected (top cycles) populations. CPR: clinical pregnancy rate

Figure 2 shows the clinical pregnancy rates by population and type of transfer, relative to the serum progesterone threshold of 0.9 ng/ml on the hCG day. We noted a significant difference above vs. below this threshold in the general population (after either blastocyst or cleaved embryo transfer) but not in the selected population. Figure 3 details the clinical pregnancy rate by serum progesterone class; it highlights the difference between the general population and the selected population and explains (at least in part) the results presented in Fig. 2. Thus, the highest clinical pregnancy rates in the selected population were observed for the 0.91–1.1 ng/ml class. The highest clinical pregnancy rates in the general population were observed in the 0.51–0.7 and 0.71–0.9 ng/ml classes after blastocyst transfer and in the 0.51–0.7 class after cleaved embryo transfer.

## Discussion

The results of our retrospective study highlighted a significantly higher clinical pregnancy rate after blastocyst transfer (33.3%) than after cleaved embryo transfer (25.3%; *p* <  0.01) in the general IVF/ICSI population; the same was true for the live birth rate (32.1 vs. 22.8%, respectively; p <  0.01). The same difference was found in Glujovsky et al.’s [9] meta-analysis of 27 randomized studies (covering a total of 4031 women or couples); the OR [95%CI] was 1.30 [1.14–1.47] for clinical pregnancies and 1.48 [1.2–1.82] for births (in 13 studies) after D5/D6 blastocyst transfer. For D5/D6 blastocyst transfer, Wang et al. [8] found an OR of 1.43 [1.15–1.78] for clinical pregnancies (in seven randomized studies) and an OR of 2.15 [1.57–2.94] for births (in four studies). Wang et al. also found that blastocyst transfer was associated with a lower miscarriage rate (OR [95%CI]: 0.51 [0.3–0.87]), relative to cleaved embryo transfer; this was not the case in our study (with values of 11.6% vs. 13.8%, respectively, in the general population) or in the meta-analysis by Glujovsky et al. (OR [95%CI]: 1.15 [0.88–1.50]) [9]. In our study, we transferred blastocysts on D5 only; this yields significantly more pregnancies than on D6, as shown by Bourdon et al.’s [18] meta-analysis (OR [95%CI]: 2.38 [1.74–3.24] for clinical pregnancies and 1.74 [1.37–2.20] for live births). Other researchers have not found any difference in pregnancy rates per transfer between blastocysts and cleaved embryos: with a serum progesterone level > 1.5 ng/ml on the trigger day, the rates were respectively 39.6 and 40.1% in Levi-Setti et al.’s study [19] and 35% vs. 39% in Demirel et al.’s study [20]. In our selected “top cycle” population, we did not observe any difference in the clinical pregnancy and live birth rate when comparing the blastocyst transfer and cleaved embryo transfer groups. Likewise, the miscarriage rates were similar: 8.3 vs. 8.0% in the blastocyst and cleaved embryo transfer groups, respectively. Any difference in embryo culture failure rates in the general and selected IVF/ICSI populations was reported in our study, whereas Glujovsky et al. [9] found a greater risk of no embryo transfer for D5 blastocyst versus D3 embryo.

In our study, the main predictive factor for pregnancy (whether after blastocyst transfer or cleaved embryo transfer) was the serum progesterone level on the eve of the trigger day and on the day itself; the levels were significantly higher among women who did not become pregnant in both the general and selected populations (*p* <  0.01 to < 0.001). Follicular phase progesterone facilitates the estrogens’ action on the pituitary gland, thus enabling the ovulatory peak of LH and FSH. It also has an essential role in decidualization of the endometrium and the opening of the implantation window via the structures or molecules that it controls (the pinopodes, insulin growth factor binding protein 1 and glycodelin levels, etc.) [10]. Hence, in a study of GnRH antagonist - FSH protocols, Van Vaerenbergh [21] reported differences in endometrial gene transcription between serum progesterone classes (≤ 0.9, 1–1.5, and > 1.5 ng/ml). Two pregnancies were found in the first class (out of 3 women), with two in the second (out of 6) and none in the third (out of 5). The largest number of gene transcription differences were observed when comparing the third group with the first group (*n* = 1388) and the second group (*n* = 819). The changes involved the serine protease PAPP-A, the IL-17 receptor, thrombospondin, and dickkopf homolog 3 (*Xenopus laevis*). Labarta et al. [22] found that a serum progesterone level > 1.5 ng/ml was associated with differences in the expression of proteins involved in cell adhesion, immunity, and growth. During the proliferative phase, progesterone levels are usually < 0.5 ng/ml, with a luteinization induction threshold of between 1 and 10 ng/ml. Filicori et al. [23] showed that the elevation in progesterone depended on the granulosa cells and (as also reported by other researchers [24–26]) was associated with the number of oocytes retrieved, the dose of FSH, and the serum estradiol level. This premature elevation led some researchers [27, 28] to recommend an earlier trigger. The elevated serum progesterone level on the trigger day affects the endometrium but not embryo quality, as shown in Huang et al.’s study [29] and by the results of frozen embryo or donor embryo transfers [14, 24, 30]. Furthermore, the very high serum estradiol level accelerates endometrial maturation (with no pregnancies if the advance exceeds 3 days [31]) and leads to luteal-phase LH deficiency, luteolysis, myometrial hypercontractility, and differences in gene transcription. Saadat et al. [32] found that the mean ± SD acceleration in endometrial maturation was the same for antagonist protocols (5.8 ± 0.4 days) and agonist protocols (5.9 ± 0.7 days) and persisted up until 7 days after the trigger.

Elevated progesterone levels before the ovulation trigger are responsible for a decrease in pregnancy rates. The research question addressed in the literature concerns the serum progesterone threshold above which this effect appears. According to Bosch et al. [33], the threshold is 1.5 ng/ml, whereas Xu et al. [26] quoted values of 1.5 to 2.75 ng/ml, depending on the follicular response. In fact, Xu et al.’s study of 10,000 cycles [26] found a fall in the rates of pregnancy above 2.75 ng/ml in strong responders (> 20 oocytes), above 1.75 ng/ml in normal responders, and above 1.5 ng/ml in nonresponders. In contrast, the pregnancy rates for frozen embryo transfers increased with the serum progesterone level. In our study, 77 couples in the selected population had frozen embryos after freeze-all (mean number of embryos frozen: 3.43 ± 2.14), which prompts us to expect even higher cumulative pregnancy and birth rates. Corti et al. [34] reported a difference in the clinical pregnancy rate above vs. below a serum progesterone threshold of 1.5 ng/ml. (50% vs 33.3%, respectively; OR [95%CI]: 2 [1.07–3.75]). Other researchers have found a lower serum progesterone threshold. Venetis et al.’s study of 60,000 cycles [14] found an OR for birth of 0.39 when the serum progesterone level on the trigger day was between 0.4–0.6 ng/ml, with 0.79 between 0.8 and 1.1 ng/ml, 0.67 between 1.2 and 1.4 ng/ml, 0.64 between 1.5 and 1.75 ng/ml, and 0.68 between 1.9 and 3 ng/ml. Venetis et al. did not observe an impact of a serum progesterone level ≥ 1.5 ng/ml in poor responders (< 6 oocytes, *n* = 796 cycles) or strong responders (> 18 oocytes, *n* = 730). In 2015, the same research group found a threshold of 0.9 ng/ml for 3296 cycles [35]. Griesinger et al. [36] found that the prevalence of an elevated serum progesterone level varied with the follicular response: 4.5% for poor responders (≤5 oocytes) vs. 19% for strong responders (> 18 oocytes). The pregnancy rate was also low (OR: 0.55 [0.37–0.81]), especially in poor responders. Fanchin et al. [37] came to the same conclusion in poor responders for a serum progesterone threshold at 0.9 ng/ml but this level did not have an impact among normal responders. Requena et al. [38] found the same pregnancy rates in strong responders (> 20 oocytes and 3000 pg/ml) above and below a serum progesterone level of 1.5 ng/ml. In our study, the serum estradiol level on the trigger day and the number of oocytes retrieved testified to a normal response to ovarian stimulation. Santos-Ribeiro et al. [39] found the best pregnancy rate (29.7%) when the serum progesterone level was between 0.76 and 1 ng/ml, after adjustment for female age, the number of oocytes retrieved, the serum estradiol level, the FSH dose, the number of embryos transferred, and the embryo stage. Wang et al. [40] studied the pregnancy rates after transfers of fresh embryos (*n* = 1455) or frozen embryos (n = 1455). They observed than when the serum progesterone level ≤ 1 ng/ml, the pregnancy rates for fresh and frozen embryo transfers were respectively of 56.4 and 54.6% in women aged ≤35 and 45.1 and 48.9% in woman over 35. When the serum progesterone level exceeded 1 ng/ml, the rate after fresh embryo transfer was lower (46.1% for age ≤ 35; OR: 1.38 [1.11–1.71], and 35.2% for age > 35; OR: 1.73 [1.34–2.24]); these values are similar to those found in the present study for the general population vs. the “top cycle” population. Papanikolaou et al. [41] studied the clinical pregnancy rate in GnRH antagonist-rFSH protocols as a function of the serum progesterone level on the trigger day. In case of transfer of embryos on D3, the clinical pregnancy rate was 37.5% when the progesterone level was < 0.73 ng/ml, 34% for the range 0.74–0.90, 30.6% for the range 0.91–1.2, 25.9% for the range 1.21–1.53, and 15.7% when the level was > 1.53 ng/ml (*p* = 0.008). Following blastocyst transfer on D5, there was no significant difference in the pregnancy rates as a function of the serum progesterone level (< 0.67: 42.9%; 0.68–0.99: 39.5%; 1–1.15: 29.3%; 1.16–1.48: 40%, and > 1.48: 41.5%). The authors concluded that the transfer of a good-quality blastocyst compensates for the high progesterone level. In 2012, the same research group [42] did not find a difference for a serum progesterone level above or below a threshold of 1.5 ng/ml in either GnRH antagonist or GnRH agonist protocols. In a study by Vanni et al. [43], the serum progesterone threshold for obtaining good-quality blastocysts was 1.49 ng/ml, and the yield was better below 1 ng/ml. Shapiro et al. [44] reported a lower pregnancy rate after fresh blastocyst transfer on D6 (relative to D5); this was probably due to transfer outside the implantation window, which is advanced by the ovarian stimulation (the pregnancy rate was the same for frozen embryo transfers). Healy et al. [45] compared D5 and D6 transfers and observed an influence of the serum progesterone level above 0.8 ng/ml. On D6, the relative decrease in the pregnancy rate was 8% when the serum progesterone level was normal and 17% when it was > 1.5 ng/ml. On D5, the best pregnancy rate was obtained when the serum progesterone level was < 1 ng/ml, with an OR [95%CI] of 0.75 [0.67–0.88] (*p* <  0.001) when considering the serum progesterone level as a continuous variable. According to Huang et al. [29], the best pregnancy rates were observed after embryo transfers on D3 or D5 when the serum progesterone level was between 0.5 and 0.74 ng/ml. Overall, 50.4% of the women became pregnant when the serum progesterone level was < 1 ng/ml, with 45.5% when it was between 1 and 1.49 ng/ml and 36.2% when it was ≥1.5 ng/ml (*p* <  0.01). In our study (with a threshold of 0.9 ng/ml), there was a significant difference in the clinical pregnancy rate between the blastocyst transfer and cleaved embryo transfer groups in the general population but not in the selected “top cycle” population (Fig. 2). Unlike Hill et al. [46] who have showed that an elevated serum P level on the day of hCG administration was negatively associated with live birth, even in good prognosis embryo transfers, but in accordance with Papanikolaou et al. [41], it appears in our study that the selection of “top cycles” reduced the impact of an elevated serum progesterone level on the trigger day. This influence can be seen in our Fig. 3, where the clinical pregnancy rates were high in the selected population up to a serum progesterone level of 1.1 ng/ml for both blastocyst transfer and cleaved embryo transfer.

Lastly, the serum progesterone level was independent of the type of gonadotropin and the duration of administration [38, 47, 48], as shown by the absence of a difference for these parameters in our study. In a report published in 2014, Lee et al. [49] showed that elevation of the progesterone level for 2 or more days was associated with a lower pregnancy rate (20.7%, vs. 39.4 for women with a normal level; *p* <  0.001). This might explain our results in the general population and the selected population, where the serum progesterone levels during the last two days of the stimulation were significantly higher in non-pregnant women (regardless of the embryo transfer stage). Nevertheless, Santos-Ribeiro et al. [50] did not find a lower birth rate when the serum progesterone level was above 1 ng/ml (even after more than a day with elevated levels before the trigger), whereas the rate fell when the serum progesterone level was > 1.5 ng/ml (30.3% in the absence of an elevated serum progesterone level, 20.4% for one day of elevation, and 20.5% for more than one day). However, a progesterone level > 1.5 ng/ml was very infrequent (1.9%) in Santos-Ribeiro et al.’s study.

We studied various ratios involving the serum progesterone level on the trigger day (Table 3): POI, P:E2, and PFI. The PFI was considerably higher in the general population than in the selected population, and considerably higher (*p* <  0.001) in the women receiving a cleaved embryo transfer (regardless of whether or not they became pregnant). Shufaro et al. [17] estimated that the PFI was more representative of follicular progesterone secretion and the cycle outcome than the mere number of oocytes collected. For 8649 cycles followed by cleaved embryo transfer, the researchers found that the mean PFI was 0.32 ± 0.25 nM, and that the chance of pregnancy was four times lower if the PFI was high (>93rd percentile, i.e. a serum progesterone level > 4.2 nM and a PFI > 0.6). The pre-ovulatory serum progesterone level was 2.22 ± 1.33 nM in Shufaro et al.’s study. The authors recommended using the PFI rather than the progesterone level on the trigger day to decide whether to continue a cycle (i.e. with a low PFI) or to abandon it (i.e. with a high PFI and, in some cases, a high serum progesterone level). In our study, the PFI was always above 0.6 (probably because of a low serum progesterone level) and was significantly higher in the selected population and in non-pregnant women after cleaved embryo transfer; these findings are in line with Shufaro et al.’s findings [17], which did not apply to cases of blastocyst transfer. The POI even differed significantly when comparing pregnant and non-pregnant women after a cleaved embryo transfer in the general population (*p* <  0.01). Furthermore, and in contrast to the PFI, the POI after cleaved embryo transfer was significantly lower in the selected populations than in the general populations – regardless of whether pregnancy was achieved. Grin et al. [15] showed that the POI was inversely correlated with the clinical pregnancy rate (adjusted OR 95%CI: 0.063 [0.016–0.249]; *p* <  0.001) and the birth rate (0.036 [0.007–0.199]; p <  0.001), and that the 90th percentile of this ratio was 0.36 ng/ml/oocyte (clinical pregnancy rate: 8%; birth rate: 5.9%). It is noteworthy that the POI was always well below 0.36 ng/ml/oocyte in our study. For the P:E2 ratio (indicating premature luteinization when > 1 [16] or ≥ 1.2 [51]), we only found a significant difference for blastocyst transfers in the general population. In Lai et al.’s study [51] of GnRH agonist protocols, a P:E2 ratio < 1.2 (mean: 0.6 ± 0.3 ng/ml) had no impact on the ongoing pregnancy rate (29.3%, vs. 34.5% for P:E2 > 1.2). The sensitivity was 75%, the specificity was 32%, the positive predictive value was 37%, the negative predictive value was 71%, and the area under the ROC curve [95%CI] was 0.534 [0.456–0.613], corresponding to poor predictive value for the P:E2 ratio in GnRH agonist protocols. In a study of women who responded strongly to a GnRH agonist protocol, Lee et al. [52] found that the highest pregnancy rates were achieved with a serum progesterone level ≤ 0.9 ng/ml or between 0.9 and 1.4 ng/ml, with mean ± SD P:E2 ratios of 0.18 ± 0.01 and 0.27 ± 0.01, respectively. It is probable that the premature luteinization threshold in Lee et al.’s study (P:E2 > 1) was low because the hyperstimulation had already contributed to an acceleration in endometrial maturation [53]. In a study of women with a serum progesterone level ≥ 1.5 ng/ml during GnRH antagonist protocols, Golbasi et al. [54] showed that the P:E2 ratio did not have predictive power (mean value: 0.73 ± 0.54 for births vs. 1.05 ± 1.38 in the absence of births; *p* = 0.158). In GnRH agonist protocols, effective blockade of LH elevation (in 95–98% of the women) meant that the elevated serum progesterone level before the trigger must have been caused by another factor. Hence, in our study (where the P:E2 ratio was always below 0.6), the LH level was higher (in 75% of cases) among women with a low serum progesterone level on the trigger day. This finding was also described by Huang et al. [29]: the mean ± SD serum LH level on the trigger day after cleaved embryo transfer on D3 was 2.0 ± 1.3 IU/l when the serum progesterone level was < 1 ng/ml and 1.7 ± 1.0 IU/l when it was ≥1.5 ng/ml (*p* <  0.01). The use of hMG (with hCG activity) for ovarian stimulation might explain these results [55]; however, we did not find a significant difference between the pregnant and non-pregnant groups in this respect.

Our study was limited by its retrospective character and its low statistical power, relative to the literature [14]. Nevertheless, our random choice of the embryo transfer day enabled us to obtain two demographically similar populations. We excluded cases in which the serum progesterone level was > 1.5 ng/ml, which probably impacted the pregnancy rates. Likewise, the high incidence of “freeze-all” cycles in each group decreased the pregnancy rate per retrieval, and prompted us to only consider the pregnancy rate per transfer. The subsequent transfer of frozen embryos should result in even higher cumulative pregnancy rates. Lastly, our use of the same quality-controlled progesterone assay throughout our study minimized the variability in the concentration data. However, inter-assay differences are frequent, and so our results may differ from those of other studies.

## Conclusions

Our present results showed that clinical pregnancy and live birth rates are significantly higher after blastocyst transfer than after cleaved embryo transfer in a general IVF/ICSI population but not in a selected “top cycle”) population. One of the predictive factors for pregnancy was the serum progesterone level on the eve of the trigger day and on the day itself, which was significantly lower in the subgroups of women who became pregnant. In our study, the selection of “top cycles” reduced the impact of an elevated serum progesterone level on the trigger day, likely because of the good embryo quality that compensates the advance of luteal endometrial maturation associated with the premature elevation of the progesterone. We therefore recommend blastocyst transfer (or, for “top cycles”, cleaved embryo transfer) in women with a serum progesterone level < 1 ng/ml on the trigger day. Further clinical, histological and genomic/proteomic studies will be required to better understand the impact of low serum progesterone levels on pregnancy rates [56].

## Data Availability

The material contained in this manuscript has not been published, has not been submitted or is not being submitted elsewhere. The datasets used and/or analysed during the current study are available from the corresponding author on reasonable request.

## Abbreviations

AMH: Anti-Müllerian hormone
ART: Assisted reproductive technology
BMI: Body mass index
95%CI: Confidence interval at 95%
CP: Clinical pregnancy
D2, D3, D5, D6: day 2, 3, 5 or 6
E2: Estradiol
FSH: Follicle stimulating hormone
GnRH: Gonadotropin releasing hormone
hCG: Human chorionic gonadotropin
hMG: Human menopausal gonadotropin
ICSI: Intracytoplasmic sperm injection
IVF: In vitro fertilization
IR: Implantation rate
LH: Luteinizing hormone
NS: Not significant
O: Oocyte
OP: Ongoing pregnancy
OR: Odd-ratio
P: Progesterone
2 PN: Two pronuclei
P:E2: Progesterone to estradiol ratio
PFI: Progesterone to follicle index
POI: Progesterone to oocyte index
ROC: Receiver operating curve
SD: Standart deviation
WHO: World Health Organization
